# Histological and immunohistochemical characterization of the inflammatory and glial cells in the central nervous system of goat fetuses and adult male goats naturally infected with *Neospora caninum*

**DOI:** 10.1186/s12917-014-0291-7

**Published:** 2014-12-14

**Authors:** Rafael Carneiro Costa, Débora Ribeiro Orlando, Camila Costa Abreu, Karen Yumi Ribeiro Nakagaki, Leonardo Pereira Mesquita, Lismara Castro Nascimento, Aline Costa Silva, Paulo César Maiorka, Ana Paula Peconick, Djeison Lutier Raymundo, Mary Suzan Varaschin

**Affiliations:** Universidade Federal de Lavras, Setor de Patologia Veterinária, Caixa postal 3037, Lavras, MG Brazil; Universidade de São Paulo, Faculdade de Medicina Veterinária e Zootecnia, Av. Prof. Dr. Orlando Marques de Paiva, 87 - Cidade Universitária, São Paulo, SP Brazil

**Keywords:** Abortion, Encephalitis, Neosporosis, PCR

## Abstract

**Background:**

*Neospora caninum* is an apicomplexan protozoan that is considered one of the main agents responsible for abortion in ruminants. The lesions found in the central nervous system (CNS) of aborted fetuses show multifocal necrosis, gliosis, and perivascular cuffs of mononuclear cells, but the inflammatory and glial cells have not been immunophenotypically characterized. The lesions in the CNS of infected adult animals have rarely been described. Therefore, in this study, we characterized the lesions, the immunophenotypes of the inflammatory and glial cells and the expression of MHC-II and PCNA in the CNS of goats infected with *N. caninum*. The CNS of eight aborted fetuses and six adult male goats naturally infected with *N. caninum* were analyzed with lectin histochemistry (RCA1) and immunohistochemistry (with anti-CD3, −CD79α, −GFAP, −MHC-II, and -PCNA antibodies). All animals were the offspring of dams naturally infected with *N. caninum.*

**Results:**

The microscopic lesions in the CNS of the aborted fetuses consisted of perivascular cuffs composed mainly of macrophages (RCA1^+^), rare T lymphocytes (CD3^+^), and rare B lymphocytes (CD79α^+^). Multifocal necrosis surrounded by astrocytes (GFAP^+^), gliosis composed predominantly of monocytic-lineage cells (macrophages and microglia, RCA1^+^), and the cysts of *N. caninum*, related (or not) to the lesions were present. Similar lesions were found in four of the six male goats, and multinucleate giant cells related to focal gliosis were also found in three adult goats. Anti-GFAP immunostaining showed astrocytes characterizing areas of glial scarring. Cysts of *N. caninum* were found in three adult male goats. The presence of *N. caninum* was evaluated with histopathology, immunohistochemistry, and PCR. Immunohistochemistry demonstrated anti-PCNA labeling of macrophages and microglia in the perivascular cuffs and the expression of MHC-II by microglia and endothelial cells in the CNS of the aborted fetuses and adult male goats.

**Conclusions:**

Macrophages and microglia were the predominant inflammatory cells in the CNS of aborted fetuses and healthy adult male goats infected with *N. caninum*. Activated astrocytes were mainly associated with inflamed areas, suggesting that astrocytes were involved in the resolution of the lesions.

## Background

*Neospora caninum* is an apicomplexan protozoan of the family Sarcocystidae [1]. Its definitive hosts are dogs (*Canis familiaris*) [2], coyotes (*Canis latrans*) [3], dingoes (*Canis lupus dingo*) [4], and gray wolves (*Canis lupus*) [5]. Neosporosis is considered the major cause of abortion in bovines worldwide and congenital infection is the main cause of its maintenance in herds [6]. Many cases of reproductive problems associated with *N. caninum* in goats have been described [7-11], but the birth of healthy and uninfected animals has also been reported [12].

The main lesions found in tissue sections of the central nervous systems (CNS) of the aborted fetuses are multifocal necroses, glioses, and perivascular mononuclear cell cuffs, together with *N. caninum* itself [11,13-15]. Similar lesions to those found in fetuses were observed in a sheep [16] and cow [17] diagnosed with neosporosis by the isolation of the parasite and PCR, respectively.

Although many cases of neosporosis have been reported in ruminants, the inflammatory and glial cells within the CNS lesions have not been characterized. Therefore, the aim of this study was to characterize the inflammatory response and the glial cells in the CNS lesions in fetuses aborted by *N. caninum* infection and in healthy male goats naturally infected with the protozoan. This is the first report of *N. caninum* cysts in the CNS of adult goats.

## Methods

The experiment was conducted in the Laboratory of Veterinary Pathology at the Federal University of Lavras (UFLA) in the state of Minas Gerais, Brazil. The study was approved by the Ethics Committee for Animal Use at UFLA, under protocol number 081/13.

### Animals

We selected 14 goats for this study from our institutional herd: six healthy adult males, aged from 6 months to 3 years, and eight aborted fetuses (90–150 days’ gestation). The goats’ dams were naturally infected with *N. caninum*, identified by the detection of specific antibodies with an indirect fluorescent antibody test (IFAT; initial serum dilution, 1:50), and seronegative for *Toxoplasma gondii* by IFAT (initial serum dilution, 1:64). The congenital infection of the adult male goats was confirmed by the detection of specific antibodies with IFAT (1:50) in sera obtained from blood samples collected before the ingestion of colostrum and by the detection in the dams’ placentas of *N. caninum* DNA with PCR and DNA sequencing. The male goats were animals scheduled for disposal that had been kept in pens since birth to avoid exposure to sporulated *N. caninum* in the environment. All the male goats were seronegative for *T. gondii* by IFAT*. Neospora caninum* infection in the fetuses was confirmed with PCR and DNA sequencing of their placentas and CNS, with the methodology described by Mesquita et al. [12]. Four fetuses and one adult male that were seronegative for *N. caninum* and *T. gondii* according to PCR and IFAT were used as the negative controls.

### Sample collection and processing

The fetuses were necropsied shortly after abortion, and the adult males after euthanasia under anesthesia with thiopental and a subsequent intravenous infusion of potassium chlorate solution. Tissue samples from all the animals were collected in 10% neutral-buffered formalin. Samples of heart, lung, kidney, liver, skeletal muscle, brain (cerebral cortex, thalamus, hippocampus, rostral and caudal colliculi, cerebellar peduncle, cerebellum, and obex), and spinal cord (cervical, thoracic, and lumbar) were processed routinely for histopathology and immunohistochemistry. The lesions were classified as discrete, moderate, or severe. Samples of the cerebral cortex, thalamus, and cerebellum were also collected and stored at −20°C for PCR analysis.

### Immunohistochemistry

To evaluate the lesions and cellular immunological response in the CNS, the following antibodies were used: anti-CD79α (Dako) for B lymphocytes; anti-CD3 (Dako) for T lymphocytes; anti-glial fibrillary acidic protein (GFAP; Dako) for astrocytes; anti-G-H42a (Washington State University) for major histocompatibility complex II (MHC-II) molecules; and anti-proliferating cell nuclear antigen (PCNA; Dako) for proliferating cell nuclear antigen, at dilutions of 1:50, 1:500, 1:1000, 1:500, and 1:1000, respectively. To confirm the presence of *N. caninum* in tissue slices, an anti-*N. caninum* antibody (VMRD, Inc., Pullman WA, USA) was used. Antigen retrieval for *N. caninum* and GFAP was performed in citrate buffer (pH 6.0), whereas Tris–EDTA buffer was used for the other antibodies; all slices were irradiated for 6 min at full power in a domestic microwave. Samples of normal CNS, lymph nodes, tonsils, and tissues that contained *N. caninum* were used as the positive controls. As a negative control, the antibody was substituted with phosphate-buffered saline. Additional brain sections from the infected animals were subjected to immunohistochemistry using an anti-*T. gondii* antibody (VMRD Inc.).

Immunolabeling was classified according to the number of stained cells in a single field at 400× magnification, as discrete (+), fewer than 10 stained cells per field; moderate (++), 10–30 stained cells per field; and severe (+++),more than 30 stained cells per field.

The immunolabeled cells in the lesions were morphologically characterized. Immunolabeled astrocytes in the unaffected areas were not considered (Tables 1 and 2).

**Table 1:** **Neosporosis in goats, lesions, diagnosis and immunolabelling in fetuses**

**Foetus**	**Age (days)**	**Lesions**			**Parasite confirmation**			**Immunolabelling**					
		**Gliosis**	**P. cuffs**	**Necrosis**	**PCR**	**HE**	**IHQ**	**RCA**	**GFAP**	**PCNA**	**MHC-II**	**CD3**	**CD79α**
1	90	+++	+++	-	+	-	-	+++	+	+++	-	-	-
2	90	-	-	-	-	-	-	-	-	-	-	-	-
3	90	-	-	-	-	-	-	-	-	-	-	-	-
4	90	+++	+++	+	+	+	+	+++	+	++	-	+	-
5	150	+	-	-	+	+	+	-	++	-	-	-	-
6	150	+	-	-	+	+	+	++	++	-	-	-	-
7	120	+	+	-	+	+	+	+	-	-	++	+	-
8	120	++	+	+	+	-	-	++	+	-	++	-	-
9*	150	-	-	-	-	-	-	-	-	-	-	-	-
10*	90	-	-	-	-	-	-	-	-	-	-	-	-

Lesions graduation: discrete (+), moderate (++) and severe (+++). Immunohistochemistry labelling graduated by the number of cells in a field of 400x: less than 10 cells per field (+), 10 to 30 cells per field (+ +), more than 30 cells per field (+ + +). *Negative Controls. P. cuffs (perivascular cuffs), HE (Hematoxilin and Eosin staining), PCR (Polimerase chain reaction), IHQ (Immunohistochemistry).

**Table 2:** **Neosporosis in goats, lesions, diagnosis and immunolabelling in adult male goats**

**Goat**	**Age (months)**	**Lesions**			**Parasite confirmation**			**Immunolabelling**					
		**Gliosis**	**P. cuffs**	**M.G.C**	**PCR**	**HE**	**IHQ**	**RCA**	**GFAP**	**PCNA**	**MHC-II**	**CD3**	**CD79α**
1	12	+++	+++	+	+	-	-	+++	+++	+	+++	+	-
2	6	+	+	-	+	+	+	-	++	-	-	+	-
3	12	+++	+++	+	+	+	+	+++	+	++	++	++	+
4	12	+	++	+	+	+	+	++	-	-	++	-	-
5	6	-	+	-	+	-	-	-	-	-	-	+	-
6	36	-	+	-	+	-	-	-	-	-	+	+	-
7*	10	-	-	-	-	-	-	-	-	-	-	-	-

Lesions graduation: discrete (+), moderate (++) and severe (+++). Immunohistochemistry labelling graduated by the number of cells in a field of 400x: less than 10 cells per field (+), 10 to 30 cells per field (+ +), more than 30 cells per field (+ + +). *Negative Control. P. cuffs (perivascular cuffs), M.G.C (Multinucleated giant cells), HE (Hematoxilin and Eosin staining), PCR (Polimerase chain reaction), IHQ (Immunohistochemistry).

### Lectin histochemistry

Biotinylated *Riccinus communis* agglutinin (RCA1; Vector), diluted 1:1000 (2 μg/ml), was incubated with the CNS samples overnight to identify the microglia and macrophages. The antigen was retrieved in citrate buffer (pH 6.0) after irradiation of the samples for 6 min at full power in a domestic microwave.

### Molecular analysis

Samples of cerebral cortex, thalamus, and cerebellum were collected and stored at −20°C until analysis. DNA was extracted from them with a commercial kit (Wizard® SV Genomic Purification System, Promega, Madison, WI, USA) after lysis with proteinase K. To detect *N. caninum*, primers based on chromosome XII of *N. caninum* were used (forward 5′-CTGTTAGAAGGTGCGGCGAA-3′ and reverse 5′-TCTCTTGCTGCGGTGGAAAT-3′), as described by Orlando et al. [18], to amplify an expected fragment of 168 bp. The PCR products were resolved by electrophoresis in 1% agarose gel at 100 V for 1 h. The amplicons of the positive samples and the positive control were quantified spectrophotometrically and sequenced with the dideoxy chain termination technique [18].

## Results

Tables 1 and 2 show the ages of the goats and the gestational stages at which abortion occurred, the occurrence and intensity of the histopathological lesions, the method of diagnosis of *N. caninum* infection, and the intensity of the cellular immunolabeling in the lesions. Fetuses 1–4 were the products of a single gestation (born to the same mother), as were fetuses 5 and 6, and fetuses 7 and 8. Fetuses 2, 3, 9, and 10, and the male goat 7, which were all negative for *N. caninum*, were used as negative controls. Goat 1 exhibited clinical neurological signs at birth, with moderate paresis, lack of coordination of the pelvic limbs, and difficulty in standing. These clinical signs had decreased a week after birth, and normal development proceeded until 12 months of age, when the animal was euthanized.

### Necropsy and histopathological findings

**Figure 1:**
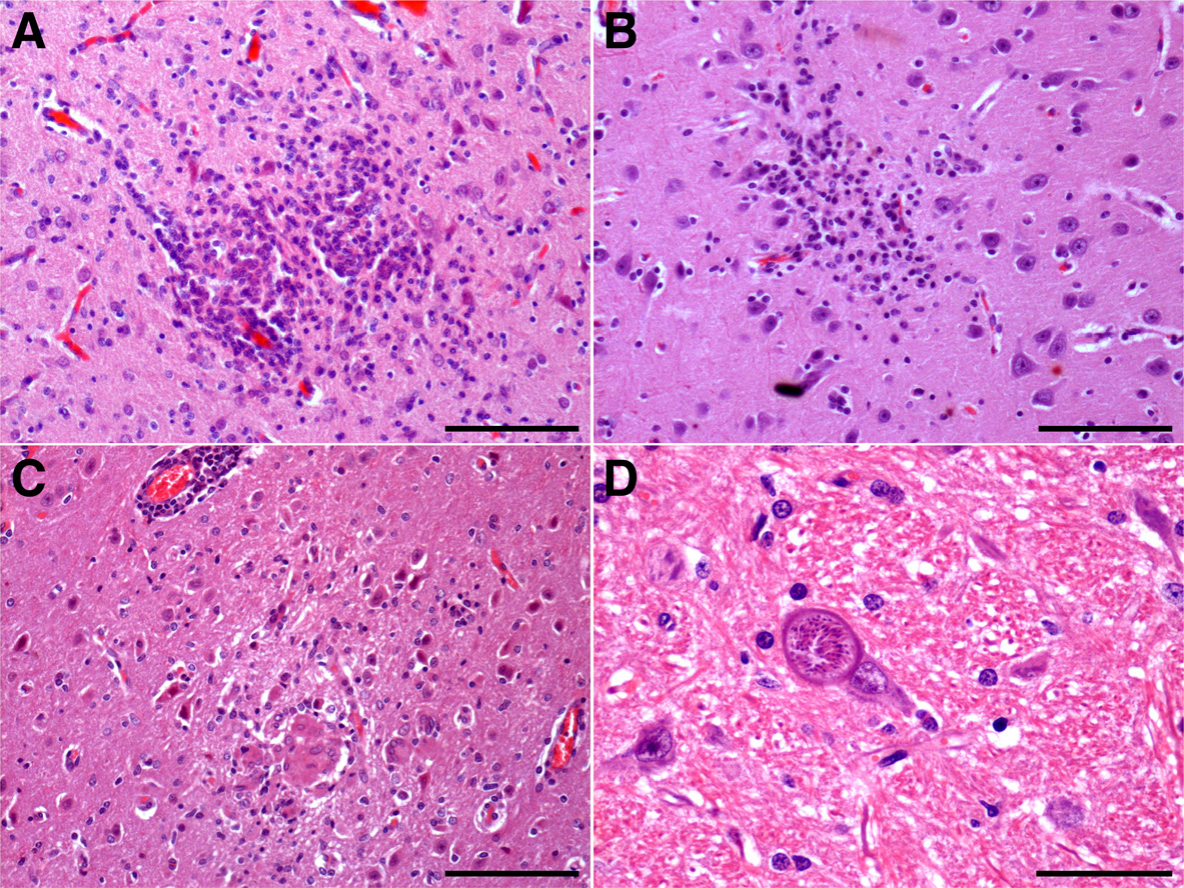
**Neosporosis in goats: central nervous system lesions in naturally infected animals.** Hematoxylin and eosin staining. **A**. Glial focus, cells with rounded and hyperchromatic nuclei in the brainstem (fetus 4); bar = 100 μm. **B**. Gliosis in the cerebral cortex, cells with rounded and hyperchromatic nuclei (goat 1); bar = 100 μm. **C**. Cerebral cortex near the cruciate sulcus, displaying multinucleategiant cells and perivascular cuffs (goat 1); bar = 50 μm. **D**. Obex. *Neospora caninum* cyst in the neuronalcytoplasm (goat 4); bar = 50 μm.

The goats exhibited no macroscopic lesions. The microscopic findings in the fetuses were discrete to moderate perivascular mononuclear cuffs (fetuses 1, 4, 7, and 8), observed near the glioses. The glioses were focal or multifocal and were observed with decreasing frequency in the cerebral cortex (fetuses 5–7), rostral colliculus (fetuses 4–6), thalamus (fetuses 4, 7, and 8), caudal colliculus (fetuses 5 and 6), medulla oblongata and obex (fetuses 1 and 4) (Figure 1A), cerebellar peduncles (fetus 4), pons, and the cervical and lumbar spinal cord (fetus 8). Foci of necrosis surrounded by glial cells and inflammatory cells were also observed in fetus 4 (cranial colliculus, pons, and thalamus) and fetus 8 (thalamus and lumbar spinal cord). Discrete mononuclear meningitis was observed close to the cerebral cortex in fetuses 4–6. *Neospora caninum* cysts were observed in the thalamus (fetuses 4–7) and the cerebral cortex (fetuses 5–7), close to areas of inflammation (fetus 4) or not (fetuses 4–7). In fetus 7, a parasitic cyst was seen in the neuronal cytoplasm. In fetuses 5 and 6, there were rare foci of mineralization associated with necrosis.

Only two of the aborted fetuses showed lesions in the myocardium and skeletal striated muscle. These consisted of varying degrees of mononuclear inflammatory infiltration, and in one fetus, some tachyzoites were observed with immunohistochemistry in samples of the heart and skeletal muscles.

The microscopic lesions in the adult goats were glioses (goats 1–4) (Figure 1B), and perivascular mononuclear cuffs in the cerebral cortex (goats 1–3), obex (goats 1, 3, and 4), thalamus (goats 1 and 3), pons, cerebellum, caudal and rostral colliculi (goat 3), and cervical, thoracic, and lumbar portions of the spinal cord (goat 3). Goat 6 displayed discrete perivascular cuffs in the meninges. Multinucleate giant cells were seen associated with a focal inflammatory response in the cerebral cortex (goats 1 and 3), pons (goat 3), and obex (goat 4) (Figure 1C). *Neospora caninum* cysts were observed in the cerebral cortex (goats 2 and 3), rostral colliculus (goat 3), obex (goat 4), cervical, thoracic, and lumbar segments of the spinal cord (goat 3), the neuronal cytoplasm in the obex (goat 4) (Figure 1D), and the cervical spinal cord (goat 3). No lesions were observed in the male goat or fetuses used as negative controls.

Two adult goats (male goats 2 and 3) had focal lymphoplasmacytic myositis in their skeletal muscles (semitendinosus and semimembranosus), but these lesions could not be associated with the parasite.

### Lectin histochemistry

**Figure 2:**
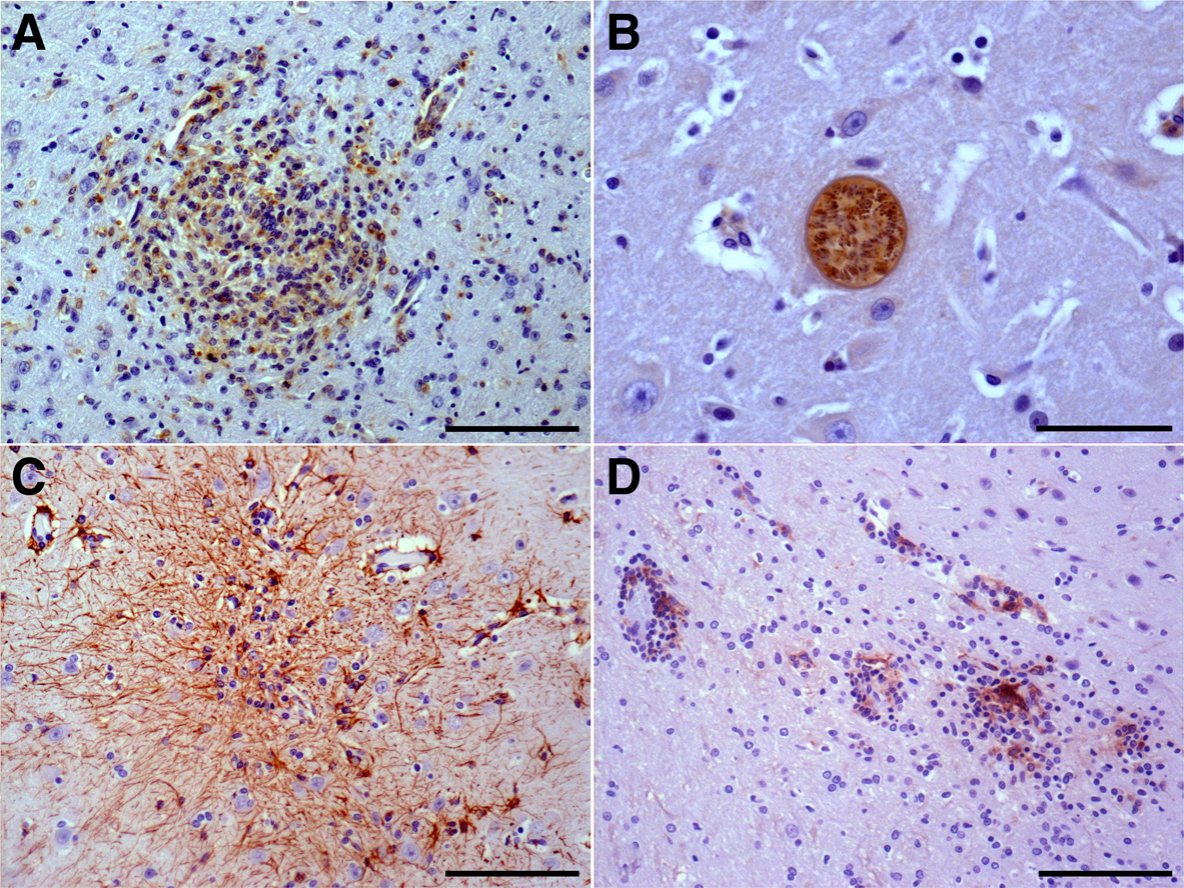
**Neosporosis in goats: central nervous system lesions in naturally infected animals. A**. Lectin histochemical staining (RCA1) revealed predominant microglia in a focus of gliosis; the same area is shown in Figure 1A (fetus 4) (streptavidin–peroxidase method); bar = 100 μm. **B**. Immunohistochemical labeling of *N. caninum*, showing a cyst in the central nervous system (goat 3) (biotin–streptavidin–peroxidase method); bar = 50 μm. **C**. Immunolabeling of GFAP in the glial focus shown in Figure 1B (goat 1) (dual link system–horseradish peroxidase [HRP] method); bar = 100 μm. **D**. Cerebral cortex: MHC-II immunolabeling in the perivascular cuff and adjacent gliosis (goat 1) (dual link system–HRP method); bar = 100 μm.

The majority of cells within the areas of gliosis were positive for RCA1. Staining occurred in the thalamus (fetuses 4, 6–8 and goat 1) (Figure 2A), cerebral cortex (fetuses 1, 4 and 7 and goats 1 and 3), obex (fetus 1 and goat 4), cerebellum (fetus 4), pons (fetus 8 and goat 3), caudal colliculus (goat 3), and the cervical (goat 3), thoracic (goat 3), and lumbar segments (fetus 8) of the spinal cord. Staining was also seen in the cells of the perivascular cuffs in the cerebral cortex (fetuses 1, 4 and 7 and goats 1 and 3), cerebellum, and cerebellar peduncle (fetus 4), pons and caudal colliculus (goat 3) obex (goat 4), thalamus (fetuses 4 and 7), and the lumbar spinal cord (fetus 8). The multinucleate giant cells seen in the male goats also stained with RCA1.

### Immunohistochemistry

#### Neospora caninum

Parasitic cysts and tachyzoites of *N. caninum* were immunolabeled in fetuses 1 and 4–7 and parasitic cysts in the adult male goats 2–4 (Figure 2B). The parasitic structures were negative for *T. gondii*.

##### GFAP

GFAP immunolabeling was observed in the cells within the glial foci in the cerebral cortex (fetuses 5 and 6), in the colliculi (fetus 4), and in an extensive area of gliosis in the cortex associated with a parasitic cyst (fetus 6), with characteristic astrocytosis (increased sizes and numbers of astrocytes) and astrogliosis (astrocyte hypertrophy: increased synthesis of intermediate filaments causing increased length and branching of the astrocytic processes). GFAP immunolabeling was also intense in the astrocytes adjacent to the glial foci in the cerebral cortex (fetus 1) and in the lumbar spinal cord (fetus 8). In the adult goats, GFAP immunolabeling occurred in the glial foci in the cerebral cortex (goats 1–3) and the thalamus (goat 1), and goats 1 and 2 displayed numerous and extremely dense astrocytic processes (glial scarring) (Figure 2C).

##### PCNA

PCNA labeling occurred in the macrophages of the perivascular cuffs in the cerebral cortex (fetus 1 and goats 1 and 3), thalamus (fetus 4), pons, caudal colliculus, and cerebellum (goat 3), and in the microglia of the glial foci in the cerebral cortex (fetus 1 and goats 1 and 3), thalamus (fetus 4 and goat 3), rostral colliculus, peduncle, and cerebellum (fetus 4), and cervical spinal cord (goat 3).

##### MHC-II

MHC-II immunolabeling occurred in the adult goats: in the cytoplasm of the endothelial cells of the meningeal blood vessels (goats 1 and 6) and the vessels of the cerebral parenchyma; in macrophages of the perivascular cuffs in the cerebral cortex (goats 1 and 3), obex (goat 4), pons, cervical spinal cord, cerebellum, thalamus, and caudal colliculus (goat 3). MHC-II immunolabeling was also seen in the glial foci in the cerebral cortex (goats 1 and 3) (Figure 2D), obex (goat 4), and the cervical spinal cord (goat 3). In fetuses 7 and 8, MHC-II labeling was observed in the glial foci, endothelia, and the perivascular cuffs.

##### CD3

Rare immunolabeled T lymphocytes were observed in the perivascular cuffs of the thalamic meninges in fetus 7, in the perivascular cuffs and foci of gliosis in the thalamus of fetus 4. In the adult goats, CD3 immunolabeling occurred in the perivascular cuffs in the meninges close to the cerebral cortex (goat 6), in the thalamic parenchyma (goat 1), and in the cerebral cortex (goats 2, 3, and 5).

##### CD79α

Rare immunolabeled B lymphocytes were observed in the perivascular cuffs and glial foci in the pons, cervical spinal cord, and thalamus of goat 3.

### PCR and sequencing

*Neospora caninum* DNA was detected with PCR in the CNS samples of the fetuses (1, and 4–8) and goats (1–6) (Tables 1 and 2) and sequenced. The nucleotide sequences showed 99.9% homology with the corresponding sequence in *N. caninum*.

## Discussion

The CNS is an immunologically privileged tissue, and the control of the immune responses there depends on the relationships between various internal factors because the blood–brain barrier restricts the migration of many cells and molecules of the immune system [19]. The gliosis, necrotic lesions, and mononuclear perivascular cuffs found in the aborted fetuses have been described previously in fetuses with neosporosis [11,13-15]. However, the gliosis and perivascular cuffs associated with parasitic cysts of *N. caninum* in the adult goats have not been described. Bishop et al. [16] described similar lesions in an adult sheep, with infection confirmed by PCR and the occurrence of protozoan tachyzoite-like structures in the vascular endothelium. However, this is the first report of cysts in the CNS of adult male goats. Sawada et al. [17] described gliosis and severe perivascular cuffs in a cow whose infection was confirmed by isolating the infective agent in cell culture.

Multinucleate giant cells were present in the CNS of the adult male goats in this study, probably associated with the phagocytosis of parasitic structures. Similar findings in an aborted goat fetus were described by Corbellini et al. [10]. Several studies of *N. caninum* infection have described perivascular cuffs, but have not described the phenotypes of the cells in those lesions [11,13-15]. In this study, lectin histochemistry with RCA1 allowed us to identify the cells in the perivascular cuffs and the glial foci, which we characterized as a monocytic lineage [20]. Anti-PCNA labeling also suggested the activation of the resident microglia in the CNS, and the possible migration of blood monocytes, corresponding to the macrophages in the perivascular cuffs.

Although RCA1 also stains endothelial cells and reactive astrocytes, when the morphologies of the cells labeled with both RCA1 and GFAP were compared, there was no doubt as to their origin (monocitic cells) and numbers.

GFAP is the most important marker of astrocytes [21]. Astrocytes were observed in the glial foci in the fetuses and adult goats, and on the borders of glioses located in the transition zone between the gray matter and white matter in two fetuses.

These findings are characteristic of astrogliosis, and demonstrate the participation of astrocytes in the lesions associated with *N. caninum* infection. This was reinforced by the observation of an agglomeration of astrocytes close to an *N. caninum* cyst. These lesions suggest glial scarring, in which astrocytes attempt to isolate a focal lesion to ensure local homeostasis in the CNS [22]. Drogemuller et al. [23] demonstrated the activation of astrocytes in *T. gondii* infections, together with the expression of a protein (gp130) that is important in the resolution of infection.

There were few labeled B or T lymphocytes in the lesions, in either the fetuses or adult animals, which could reflect the incomplete activation of lymphocytes in the CNS, which probably culminated in their rapid destruction through apoptosis [19].

The expression of MHC-II molecules in the CNS was clearly established in the adult goats and in one fetus. The presence of the parasite in the CNS probably triggered the inflammatory response that stimulated the expression of MHC-II molecules by endothelial cells and activated the microglia in the CNS. This was probably mediated by interferon γ, in accordance with the theory proposed by Aloisi et al. [19]. These findings suggest that a predominantly Th1 immune response was induced against the parasite. Becher et al. [20] proposed that activated astrocytes in CNS lesions express MHC-II molecules, but this was not observed in the present study.

Another important finding was the occurrence of encephalitis, sometimes severe and with focal granulomatous inflammation, associated (or not) with the parasitic cysts in the CNS of clinically healthy adult goats.

## Conclusion

Our results show that macrophages and microglia were the predominant inflammatory cells in the CNS of aborted fetuses and healthy adult male goats infected with *N. caninum*. Activated astrocytes were mainly associated with inflamed areas, suggesting that astrocytes were involved in the resolution of the lesions.
